# A case of haemoptysis in a girl with Noonan syndrome

**DOI:** 10.7196/AJTCCM.2020.v26i3.023

**Published:** 2020-10-13

**Authors:** R K Mopeli, M Lebea, C Verwey

**Affiliations:** Department of Paediatrics and Child Health, Faculty of Health Sciences, University of the Witwatersrand, Johannesburg, South Africa

**Keywords:** Noonan syndrome, haemoptysis, polyps, bronchial artery

## Abstract

Noonan syndrome (NS) is an autosomal dominant condition affecting 1 in 2 000 live births. It is characterised by distinctive physical features, congenital heart disease and multiple other comorbidities including haematological abnormalities. Haemoptysis is the expectoration of blood originating from the lower respiratory tract. It is uncommon in children but can be life threatening.Perfusion of the lower respiratory system arises from the pulmonary arterial circulation and the bronchial circulation, or bleeding may arise from either. In children, the most common causes of haemoptysis are respiratory tract infections, aspirated foreign bodies and bronchiectasis. We present a 7-year-old girl with recurrent haemoptysis.

## Background

A 7-year-old girl with Noonan syndrome (NS) presented with a history of recurrent haemoptysis. She was diagnosed with pulmonary stenosis at the Paediatric Cardiology Department at Chris Hani Baragwanath Academic Hospital (CHBAH) and was treated with a transannular patch at the age of 6. She had severe pulmonary regurgitation following the surgery and was on treatment with furosemide, potassium chloride and digoxin.

She presented to her local hospital with a history of coughing up fresh red blood a year after undergoing surgery. The parents could not quantify the amount of blood; however, it was thought to be significant because she had a haemoglobin level of 7.5 g/dL and required a transfusion of packed red blood cells. It was reported that she had two other episodes of haemoptysis 11 months prior but did not seek medical attention. She was airlifted to CHBAH for further management. On arrival, she was noted to be in respiratory distress and was put on 60% O_2_ via a rebreathing mask. She was pink and well perfused. She had an early diastolic murmur of pulmonary regurgitation.

**Fig. 1:**
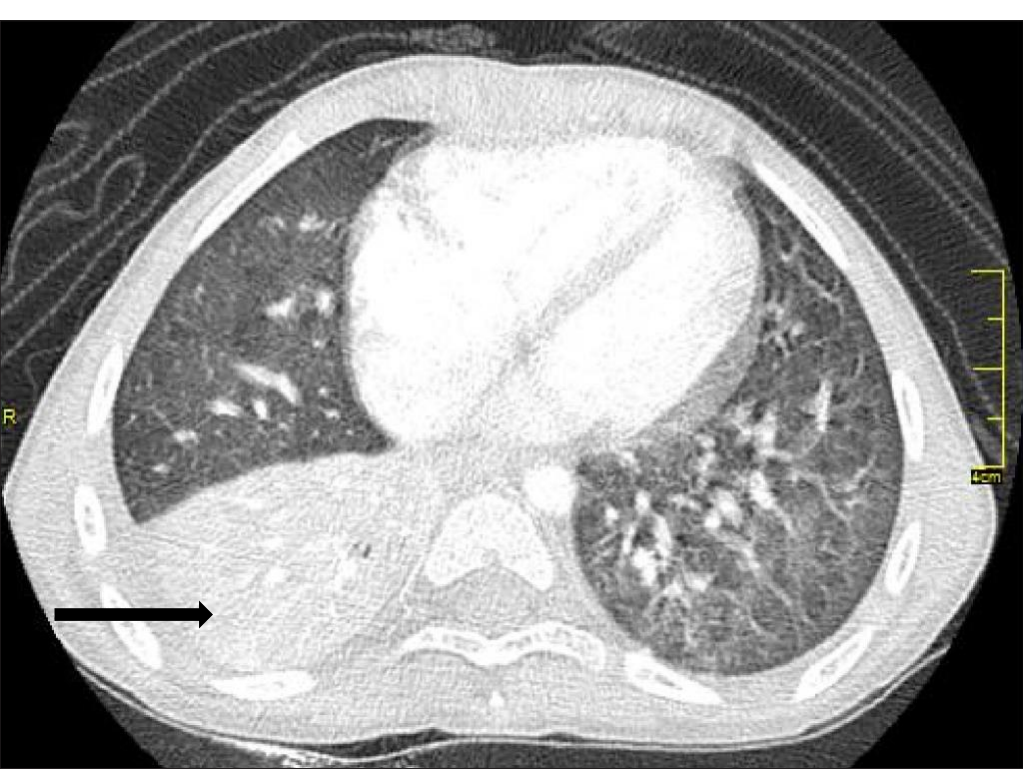
Chest computed tomography scan showing a right lower-lobe collapse consolidation (indicated by arrow).

**Fig. 2:**
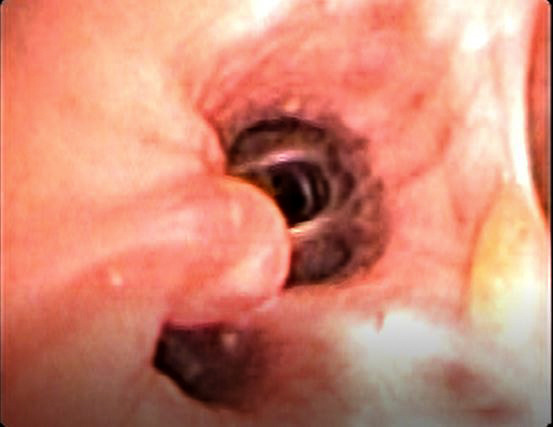
Polyp obstructing bronchial opening.

Detailed echocardiography excluded pulmonary hypertension and pulmonary vein stenosis. Laboratory investigations showed haemoglobin (11 g/dL), platelets (235 × 10^9^ cells/L), international normalised ratio (1.02) and prothrombin time (34). She was HIV-negative and had normal urea and creatinine levels. She had a von Willebrand factor antigen of 34% (normal range 50 - 160%) and activity of 88% (normal range 66 - 99%) and her Factor VIII level was 52 IU/dL. Her sputum showed no acid-fast bacilli, a *Mycobacterium tuberculosis* culture was negative and no other pathogenic bacteria were isolated. A computed tomography (CT) scan of the chest showed a right lower-lobe dense consolidation, which was thought to be due to lobar pneumonia (Fig. 1). She had a rigid bronchoscopy, which also showed an inflammatory polyp at the entrance to the posterior basal segment of the right lower-lobe bronchus (Fig. 2). The inflammatory polyp and lobar pneumonia were thought to be the cause of the haemoptysis, which was exacerbated by her underlying haematological abnormalities. She was treated with antibiotics and discharged home. A repeat bronchoscopy 3 months later showed that the polyp had significantly decreased in size.

**Fig. 3:**
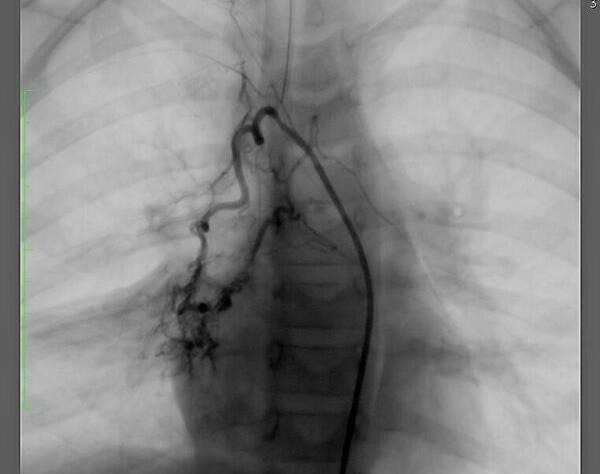
Angiogram showing the torturous right bronchial artery (Indicated by arrow)

She presented to the local hospital 2 weeks after the repeat bronchoscopy with another episode of massive haemoptysis, where she was resuscitated with fresh frozen plasma and a platelet transfusion. She was airlifted to CHBAH after stabilisation. A repeat bronchoscopy was done, but the patient had massive haemoptysis during the procedure and required resuscitation and intensive care admission for mechanical ventilation. She was referred to the cardiothoracic team for lobectomy to control the haemoptysis. She continued to have more episodes of haemoptysis while awaiting surgery. Rigid bronchoscopy was done and did not reveal a polyp. She had a cardiac catheterisation for further investigation of the haemoptysis and an angiogram showed a torturous right bronchial artery, with extravasation of blood into the right lower lobe. The patient was referred for embolisation of the torturous bronchial artery. A descending aorta angiogram in the anterior-posterior view confirmed the findings (Fig. 3). The prominent bronchial artery formed a confluence with an abnormal vessel that arises from the right common carotid artery

She had a right bronchial artery embolisation and was stable post procedure. That evening she bled again during rigid bronchoscopy. The decision was taken to do a lobectomy to control the bleeding. The histology of the resected lobe showed normal lung parenchyma, with patchy bronchopneumonia, alveolar haemorrhage and fibrointimal hyperplasia of the hilar vessels. The bronchus at the resection margin was normal and patent. There was no granulomatous inflammation or malignant neoplasm. The patient had an uneventful course post lobectomy, and after 6 months follow-up there was no further bleeding reported.

## Discussion

The diagnosis of the cause of haemoptysis in children is not easy. When investigating the aetiology, it may be helpful to divide the causes into those arising from parenchymal diseases and those arising from pulmonary vascular disorders.

It is best to begin with a detailed history and physical examination to differentiate between extrapulmonary bleeding such as haematemesis and haemoptysis. A history of foreign-body aspiration should be elicited if present. The respiratory examination may reveal localised wheezing suggestive of a foreign body or crepitations with decreased breath sounds, which may be caused by pneumonia or bronchiectasis. Radiological investigations such as a chest radiograph or CT scan will provide useful information but may also be normal. Blood tests should be done in all children to screen for anaemia, raised infective markers and bleeding abnormalities. Sputum should be evaluated for the presence of pathological microorganisms. If all the above does not lead to a diagnosis or the bleeding persists, a bronchoscopy is indicated.^[1]^

Our patient had NS which we had to take into consideration while investigating the cause of her haemoptysis. NS is a condition inherited in an autosomal dominant pattern and is present in 1 in 2 000 live births. It is characterised by distinctive physical features such as hypertelorism, low-set, backward rotated ears, a short webbed neck, short stature and multiple comorbidities including cardiac and haematological abnormalities.^[2]^ More than 80% of patients with NS have an abnormality of the cardiovascular system, with pulmonary valve stenosis and septal defects being common.^[2]^ Bleeding disorders have been reported in up to 65% of patients with NS.^[3,4]^ A number of coagulation factor deficiencies, von Willebrand disease, thrombocytopenia, and platelet dysfunction have been described.^[3,4]^ The most common factor deficiency is factor XI, followed by factors XII and VIII. Our patient was found to have von Willebrand disease. This may have worsened the degree of her blood loss, although it was not the primary cause of her bleeding. It was also clear that the polyp that had been visualised initially was not the primary cause of bleeding, as she subsequently had further haemoptysis even after it had disappeared. It could be argued that the polyp predisposed her to the lobar pneumonia, which increased vascular pressure in that area and caused the abnormal vessel to bleed.

Intracardiac left-to-right shunts can cause pulmonary hypertension in children, resulting in haemoptysis as a complication. Our patient had an echocardiography which excluded left-to-right shunting. Cardiac catheterisation did not demonstrate pulmonary hypertension in this patient, with an invasive mean pulmonary pressure of 8 mmHg. There is not much in the literature linking NS to primary abnormalities of the pulmonary vasculature. The first description of an association between NS and primary abnormalities was reported in 1989 in a 19-year-old female who had severe pulmonary hypertension, with the clinical and pathological features that were suggestive of primary pulmonary hypertension.^[5]^ Our patient was bleeding from a prominent torturous bronchial artery. This may just be an incidental abnormality aggravated by the lobar pneumonia and not necessarily related to the fact that she has NS. It could also have been part of a collateral bronchial circulation.

## Conclusion

This case illustrates the importance of having a broad-based approach when investigating any patient with haemoptysis and that many different pathologies can act together to cause haemoptysis.
